# Small Worlds and Semantic Network Growth in Typical and Late Talkers

**DOI:** 10.1371/journal.pone.0019348

**Published:** 2011-05-11

**Authors:** Nicole Beckage, Linda Smith, Thomas Hills

**Affiliations:** 1 Department of Cognitive Sciences, University of California Irvine, Irvine, California, United States of America; 2 Psychological and Brain Sciences, Indiana University Bloomington, Bloomington, Indiana, United States of America; 3 Department of Psychology, University of Basel, Basel, Switzerland; University of Maribor, Slovenia

## Abstract

Network analysis has demonstrated that systems ranging from social networks to electric power grids often involve a small world structure-with local clustering but global ac cess. Critically, small world structure has also been shown to characterize adult human semantic networks. Moreover, the connectivity pattern of these mature networks is consistent with lexical growth processes in which children add new words to their vocabulary based on the structure of the language-learning environment. However, thus far, there is no direct evidence that a child's individual semantic network structure is associated with their early language learning. Here we show that, while typically developing children's early networks show small world structure as early as 15 months and with as few as 55 words, children with language delay (late talkers) have this structure to a smaller degree. This implicates a maladaptive bias in word acquisition for late talkers, potentially indicating a preference for “oddball” words. The findings provide the first evidence of a link between small-world connectivity and lexical development in individual children.

## Introduction

The study of the dynamics and structure of complex systems has led to particularly important insights about the relations among system growth, structure and function. For example, the growth pattern of connections on the World Wide Web (the product of links from one page to another) creates hub-like patterns of connectivity that support the information and social functions of the web. Growth, structure and function may all be understood in terms of a set of related mathematical properties within the framework of network analysis [1]. Network analyses have been used to understand such wide ranging topics as the attendance of southern women at social events [2], citation networks of published scientific articles [3], and neuronal networks within the human brain [4].

Within psychology and linguistics, network analyses have been widely applied to human semantic knowledge [5]–[9]. These analyses have related structural properties of spoken language, known to be integral to language production and processing, to the hypothesized growth processes that create the network [9] [11] [12]. Analyses of the relations among semantic network growth, structure and function provide a new opportunity for understanding how typical and atypical growth patterns may relate to functional language processing. This is the focus of the present paper.

Our starting point is the finding that mature semantic networks possess small world properties [6] [9] [13]. These properties allow for high amounts of local structure combined with global access. Within these semantic networks, there is considerable local structure in the form of clusters of words that are highly interconnected to each other by semantic relatedness, which may be related to category representations [14]. However, even with these dense clusters, some words act as bridges and possibly as hubs, connecting semantically distant clusters to each other. This global access is understood as providing easy transitions from one cluster to another and is believed to support online language processing and comprehension [6] [15].

Although this functional network structure has been hypothesized to be related to the growth of semantic networks [9] [11] [12], there is no direct evidence. Nor do we know if efficient structural properties of connectivity play a role in lexical acquisition in children. One indicator that there might be a relation between early lexical acquisition and semantic network structure is that the age of acquisition of individual words is related to the semantic structure of child-directed speech as well as the connectivity pattern of words within mature language [9] [11] [12].

This prior work on age of acquisition for particular words and their role in semantic networks has used normative data on the words children typically know at a given age and have created networks by connecting words in normative vocabularies according to semantic relatedness as indicated in corpora collected from written or spoken language, free association data, or hand-coded collections of words [9] [11] [12]. As such, these analyses of developmentally early networks reveal at best the structure of the semantic system for some generic language user, but not for any individual user. To determine whether structure might be related to acquisition, the present work examines, for the first time, the structure of individual children's semantic networks and the relation between individual differences in that structure and individual differences in rate of lexical development.

The methodological approach we take is to examine the structure of the semantic networks of children who have typical or very small vocabularies for their age and gender. We took this approach because past research shows that vocabulary size at any point in development is a strong predictor of the future lexical growth rate even in children with no known developmental disorders [16] [17] [18] [19]. Indeed, a vocabulary size that is below the 20th percentile for age and gender is a marker of significant risk for future difficulties in language learning and processing [19] [20] [21] [22] [23] [24]. In the child language literature, these at-risk children are often called “late talkers”, a term that we will employ here because of its common use, although it is somewhat of a misnomer. These children often start talking later than the norm but the key fact is that they show a slower rate of vocabulary growth and smaller vocabulary sizes for their ages even after controlling for their initial delay: a difference that often persists and predicts continued difficulties in language processing tasks at least into late childhood [19] [20] [21] [22] [23] [24]. Thus, typical and late talkers show very different rates of vocabulary growth. *Are these differences in growth rates related to differences in the structure of their early semantic networks?*

## Methods

We compared the vocabularies and corresponding semantic networks for 66 children (aged 15 to 36 months), whose vocabulary sizes were either typical for their age (n = 39) or whose vocabulary sizes were small for their age (n = 27).

Children's vocabularies were collected via a widely used parent checklist, the communicative development inventory (infant form), a reliable and valid measure of lexical development [25] [26]. Each child's semantic network was derived from the list of words that parents reported their child to use in everyday speech. To construct each child's network, we connected the words in each child's vocabulary according to the co-occurrence statistics of the words in a normative language learning environment. Specifically, we used the co-occurrence statistics in the CHILDES corpus [27]-a large database of approximately 2 million words of child-directed speech targeted at English speaking children ranging in age from one to four years old, for further details see [12]. Thus, the nodes in each child's network were the words known by that child, and the links between nodes reflected the semantic relations in the language generally and were not specific to the individual child's learning environment. The resulting networks were constrained in size and content to the words asked about on the questionnaire. This consisted of 291 total words (after removing routine and timing words), of which 204 were nouns, 51 were verbs and 36 were other types of speech. We chose co-occurrence statistics as the index of semantic relatedness because they are an objective measure that aggregates semantic, conceptual and syntactic relatedness [28] [29]. They have also been shown to predict language acquisition in previous work [12]. Thus in the networks, word A was connected to word B if it appeared within the first five words that followed word B, yielding a directed network in which a relationship of A to B does not imply B is also connected to A. The window size of five was based on prior work that investigated predictive power across multiple window sizes [12].

For each individual child's network, we computed three network statistics averaged over all words in the network. These were in-degree, clustering coefficient, and geodesic distance. In-degree was computed as the number of unique word types preceding a given word in the corpus; i.e., the number of edges in the network that point towards that word. The clustering coefficient of a node, *ci* , was calculated by determining how many connections exist between nearest neighbors of that node (node *i*). The number of possible connections that can exist between neighbors is determined by the node's degree, *ki*, as follows: 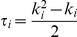. The clustering coefficient is then the fraction of observed connections, 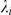, among those possible: 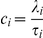. This value was averaged across all nodes to get a mean clustering coefficient for the entire network. The clustering coefficient is a common measure of local structure within the network. Geodesic distance was computed as the average shortest path length between any two nodes, and is a measure of the global access of words in the network. Together these three statistics provide three different levels of information about network structure. It should be noted that these statistics were not independent with correlation coefficients for random acquisition graphs ranging from 0.60 for in-degree and clustering coefficient to 0.76 for in-degree and geodesic distance. For a review of network analysis generally and these statistics in particular see [1] [30], and for use in semantic networks see [5] [9] [14].

Because the structure in any individual child's network cannot be meaningfully interpreted without knowing how much structure is provided by the language being learned, we first generated, for each child, a sample of 300 *random acquisition networks* that contain a randomly selected set of *n* words (where *n* is equal to a given child's vocabulary size). The words were randomly selected from the possible 291 words of the parent checklist and thus the possible words in any child's network. The edges between these randomly selected words were then constructed from the same CHILDES co-occurrence matrix that was used to provide edges in the network of an individual child. For each child, the resulting structural statistics were averaged over the 300 random networks. These random acquisition networks include the structure in the language-learning environment but remove the network structure created by the child's language sampling process as they learn words.

These random acquisition networks were then compared to 100 Erdős-Rényi random graphs (ER-graphs; [31]) produced for each individual child's vocabulary. These ER graphs contained the same number of vertices as the child's network and the same number of edges as the random acquisition network, but removed both structure inherent in the language learning environment and structure characteristic of a particular child's vocabulary. The random acquisition networks include the structure in the learning environment but remove any ordered growth patterns. The comparison of these networks to random graphs of the same size provides a direct measure of the structure due to the connections among to-be-learned words in the learning environment that is independent of the specific words learned by any child, and thus independent of the growth pattern itself.

## Results

### What would the semantic structure look like for random language learners?

**Figure 1:**
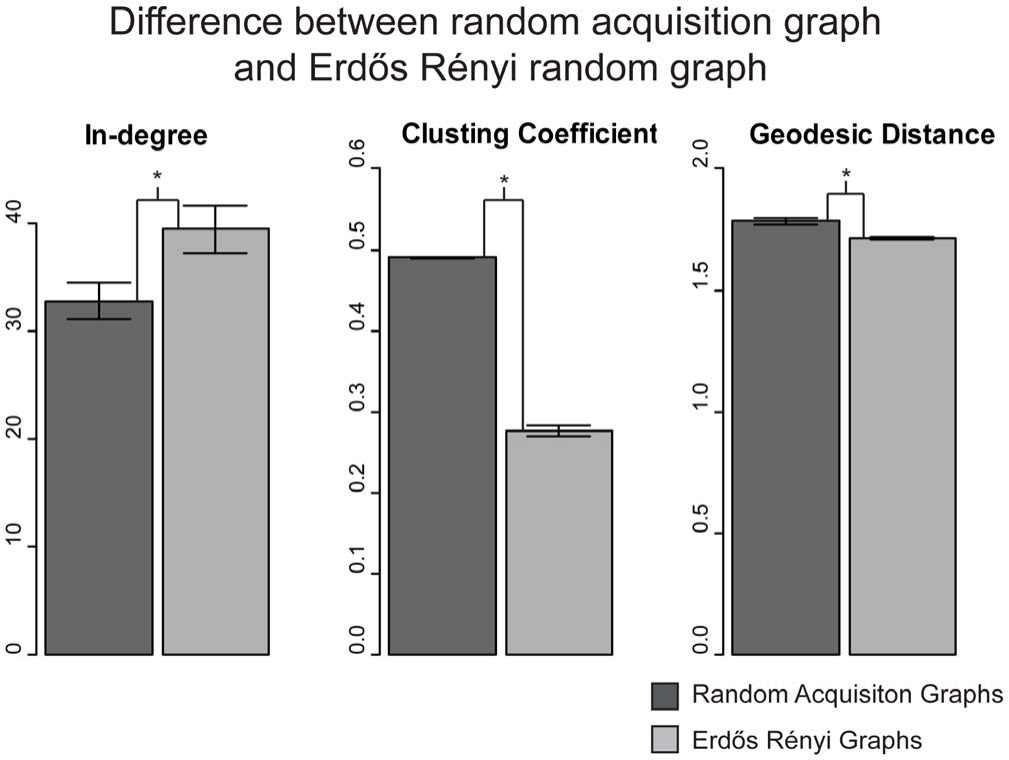
Random acquisition networks acquired from the learning environment show different structure from comparable Erd*ő*s Rényi (ER) random graphs. When semantic relatedness was used to provide connections between words, ER networks showed less clustering and lower median in-degree than random acquisition networks even when matched for density, indicating that the language environment is more structured than its random equivalent. To generate samples of random acquisition graphs, we sampled 100 random sets of words equal in number to each child's vocabulary size. Edges in the random acquisition networks were based on the same co-occurrence data as was used to build the networks of individual children. This was then averaged to get a single set of network statistics representing random acquisition for a given child. ER networks were built to contain the same overall number of edges as the averaged random acquisition graph, with 100 repetitions at the level of the individual child. All pairs are significantly different (p<.0001) with dark grey representing the ER random graphs and light grey representing the random acquisition graphs.

Figure 1 shows that the random acquisition graphs have significantly more local structure than the randomly generated ER-graphs, as indicated by the higher clustering coefficient. By design, both the random acquisition graphs and the ER-graphs have the same number of edges and nodes, but the distribution of the edges in the random acquisition graphs which is based on randomly selected words but structured connections favors local clustering in which words are often connected to their neighbors whereas the ER graphs, based on random connections among the same number of nodes, do not. This increased local structure does not harm the global structure relative to the ER graphs: the average geodesic distance, while significantly larger for the random acquisition graphs than the ER-graphs, is still similar to the ER-graph, with ER-graphs having an average shortest path length of 1.61 as opposed to the average path length of 1.78 for random acquisition networks. The random acquisition networks also have higher median in-degree, suggesting that some nodes may be acting as hubs. This indicates that the random acquisition network (from here on referred to as just random network) may have a degree distribution trending toward a scale free network (as compared to the ER network which results in a Poisson degree distribution) [32]. In brief, the language-learning environment as revealed by a large corpus of child-directed speech is so structured that if learning merely consisted of adding words selected at random, it would yield lexical connectivity with local structure and global access. This randomly acquired structure, arising from the natural structure of speech, is sufficiently strong, such that random sampling of quite small numbers of words from a corpus of two million words exhibits this small world structure. In brief, for networks of early-learned words, a small world structure does not depend on a growth pattern that preferentially acquires some words before others.

### Do typical and late talkers have the same semantic network structure?

**Figure 2:**
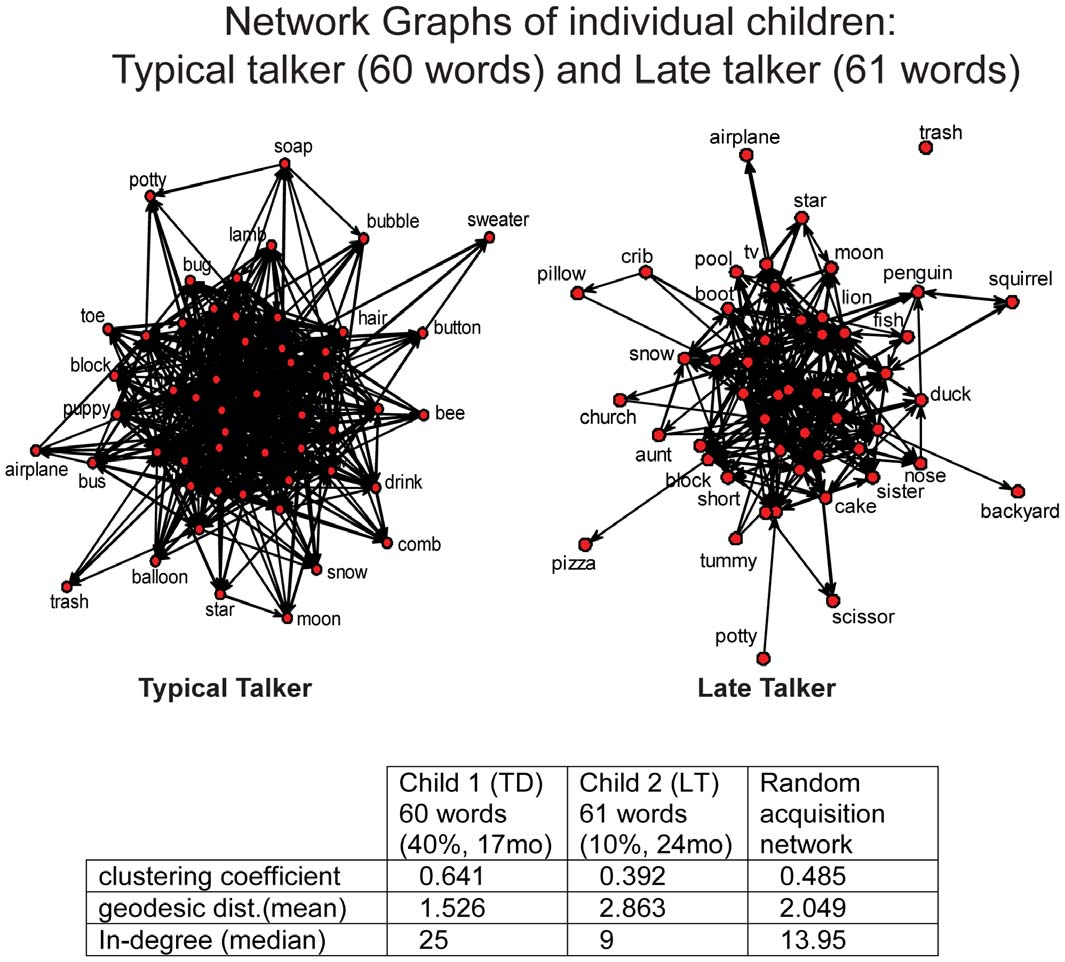
Network graphs for two individual children. The graph on the left is a typically developing (TD) child (17 mo, 40%) and the graph on the right is of an at-risk, late-talker (LT) (24 mo, 10%). The network of the TD child includes the 60 words in the child's productive vocabulary and the network of the at-risk LT child includes the 61 words in the child's productive vocabulary. The apparent visual differences in the networks are supported by the differences in the corresponding table, with the typical talker's network showing higher clustering coefficient and higher median in-degree, but lower geodesic distance, than the LT. These differences are consistent at both the individual and population level.

**Figure 3:**
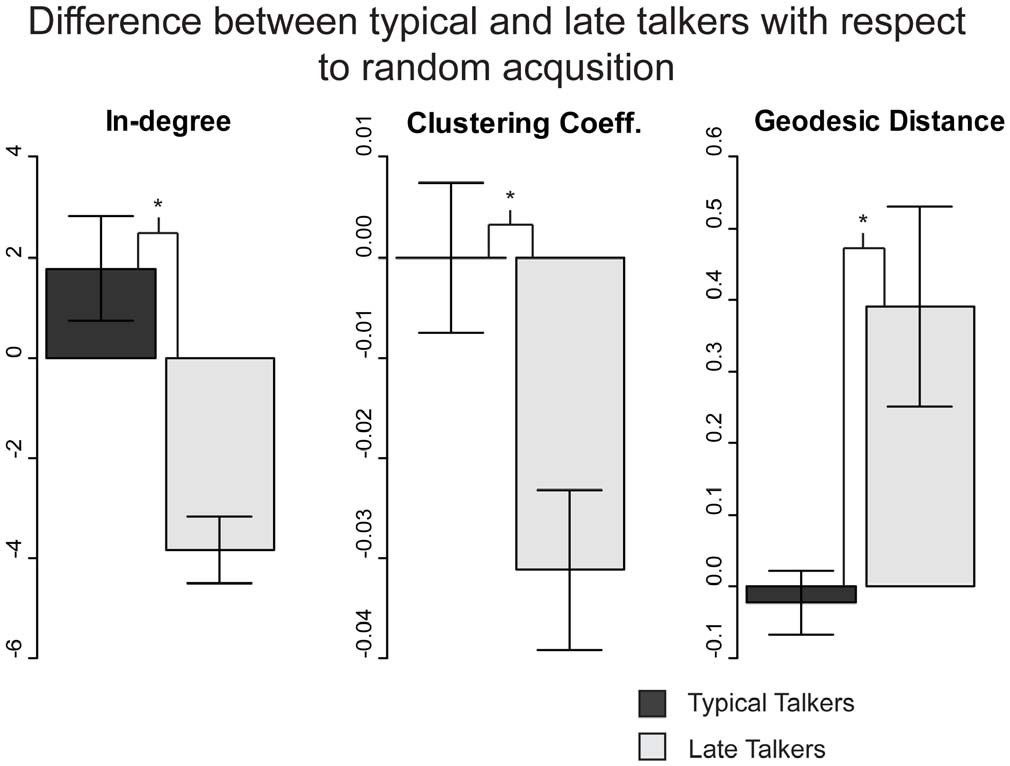
Ratios of differences relative to the child-matched random acquisition networks for both typical talkers (TD, dark gray) and late talkers (LT, light grey). TD children show marginally significant (p = 0.0970) effects of greater median in-degree and LT children show significantly less (p<0.001) median in-degree than the random acquisition networks. While TD children have clustering coefficients indistinguishable from random acquisition, the late talkers have significantly less (p<0.001) clustering than their paired random acquisition networks. TDs have no significant difference between their paired random acquisition networks whereas LTs have a significantly higher (p = .0092) geodesic distance. Error bars indicate standard error.

The conservation of this small world structure in the learning environment is not, however, observed in both typically developing children (TD) and late talkers (LT). While TD children's networks show the same structural properties as the random networks, the LT children show much less small world structure than their random acquisition networks. This suggests that LTs are sampling from the language-learning environment in such a way as to lose some of the structure inherent in language. Figure 2 shows two child networks, one from a TD child and one from an LT child, with similar vocabulary sizes. The two networks are very different in structure: the TD child's network shows more connectivity (in-degree), more local structure (clustering coefficient) and more global access (geodesic distance) than the LT child's network. These differences characterize the data from TD and LT children in general as summarized in Figure 3. Unless otherwise noted significance was measured based on a paired t-test in which each individual was compared to the average network statistics of their size-matched random acquisition graphs. Other analyses were conducted such as a one-sample t-test for each individual, with similar results. The paired t-test offered a statistical method in which individual and cross group comparisons could be conducted. Using this analysis, we found that the LT networks had significantly lower median in-degree (t(df) = 29, paired, *p*<0.001) than the corresponding size-matched random acquisition networks, whereas the TD networks did not differ significantly from their size-matched random networks (t(df) = 37, paired, *p* = 0.097). Likewise, the clustering coefficients for the LT networks were smaller than those from the corresponding random networks (t(df) = 29, paired, *p*<0.001): again the TD networks did not differ from the random networks (t(df) = 37, paired, *p* = 0.99). Further, the LT networks showed significantly lower clustering than the networks from the TD children with vocabulary size controlled (linear model, *p* = 0.003, adjusted *R*2 = 0.14, f(df) = 65). In brief, the LT networks are less connected and have less small world structure than the random networks and the TD networks.

The networks derived from the LT children's vocabularies also have less global access than the random networks and the TD children's networks. In general, as small world networks grow larger, more connections are possible and the geodesic distance (i.e shortest distance between two nodes) trends to smaller values [13]. In the randomly generated language networks and in the vocabularies from typically developing children, a low geodesic distance (less than 2) is evident in all vocabularies with at least 75 words. In contrast, even with more than 100 words, some LT networks have average geodesic distances much greater than 2. They also show significantly higher geodesic distances than their random acquisition graphs (t(df) = 29, paired, *p* = 0.009). The effect remains when comparing late talkers and typically developing children in a linear regression controlling for vocabulary size (linear model, *p* = 0.007, adjusted *R*2 = .12, f(df) = 65) Thus, the path length, a potentially important factor in language processing, is greater in the vocabularies of LT children-for any given vocabulary size-than a random sampling of words would predict or when compared to the vocabularies of their TD peers. It should also be noted that there is a significant interaction between word type (nouns, verbs, and other words) and talker type (p = .04), which is driven by typical talkers having a lower proportion of other words (non-noun and non-verb words) than late talkers (*M* = 0.08 versus 0.11, respectively; post-hoc t-test, *t*(67) = 2.62, *p* = 0.01). However, the results presented in Figure 3 were not statistically different if networks were composed of only nouns and verbs. Thus, the difference in network structure between typical and late talkers does not appear to be driven by differences in the distribution of word types that children know but instead by underlying structural differences.

## Discussion

These results show that properties of lexical connectivity that are believed to facilitate language processing are evident in the quite small vocabularies of very young children and thus may be playing a role in word acquisition and language processing at the start of language learning. The results also show that these properties characterize individual semantic networks, not just normative ones [11] [12], and that small-world connectivity in individual children's semantic networks is related to the child's rate of lexical development: Children who are building their vocabularies at the normative pace have vocabularies with small world properties; whereas late talkers-who are likely to show later language processing difficulties-show this small world structure to a much smaller degree.

These results also raise new questions about the nature of the growth processes that lead to the connectivity patterns in semantic networks. Based on the observation of small world structure, Steyvers and Tenenbaum [9] proposed a growth mechanism using preferential attachment, whereby high-connectivity in the known network selected the entry of new information from the learning environment, see [10] for a review. In contrast, the present results suggest that even random word learning would yield small world structure. If this is so, then the key open question is why children who are slow in learning language do not acquire sets of words that exhibit small world structure, like typically developing children. There are at least three possibilities. On average, random selection may yield vocabularies that exhibit small world structure, but some randomly selected sets of words will not. Thus it is possible that all children are more or less randomly selecting words, but, by chance, some children select a poorly structured set and become late talkers as a consequence. This seems unlikely given that normative evidence indicates children are not selecting words at random [11] [12]. Moreover, delays in lexical learning persist and a random selection process would be self-correcting over time.

A second possibility is that the language learning environments for late talkers are somehow distorted, exhibiting different structural properties than the environments of typical talkers. It is known that parents of children with language delay adjust their language to fit the developmental level of their child, and language delay runs in families; however, there is also other evidence to suggest that parent language per se is not the key cause of language delay in these children [26] [33]. In brief, this is a possibility that needs to be tested via network analyses of the structure of language directed to individual children.

The remaining possibility is that children with language delay sample the language learning environment in a way that limits acquisition of semantically related words [11] [12], e.g., by biased acquisition for words that are particularly novel (i.e., “oddballs”) relative to words that they already know. Thus, for example, a child using an ‘oddball’ strategy would be more likely to learn the word “telephone” than “dog” after learning the word “cat”, because “telephone” is less semantically similar to the word “cat”, which the child already knows. Formally, this would be the exact opposite of the lure of the associates model [12] to explain typical development. For this “oddball” model, words would be learned based on the lack of associative connections to the already known words as opposed to being primed for learning by associative connections; a sign change in the sensitivity parameter of the lure of the associates model would create this difference. Importantly, the lure of the associates model has been shown to predict early language acquisition for typical talkers [12]. Future analyses will need to look at the longitudinal progression of word acquisition in typical and late talkers to determine whether or not words are acquired differentially for these populations, depending on what words are already known.

## References

[1] Newman MEJ (2003). The structure and function of complex networks.. SIAM Review.

[2] Davis GF, Gardner BB, Gardner MR (1941). Deep South..

[3] Seglen P O (1992). The skewness of science.. Journal of American Society for Information Science.

[4] Sporns O (2002). Network analysis, complexity and brain function.. Complexity.

[5] Borge-Holthoefer J, Arenas A (2010). Semantic networks: Structure and dynamics.. Entropy.

[6] Cancho RF, Solé RV (2001). The small world of human language.. Proceedings of the Royal Society of London, Series B: Biological Sciences.

[7] Collins AM, Loftus EF (1975). A spreading-activation theory of semantic processing.. Psychological Review.

[8] Collins AM, Quillian M (1969). Retrieval time from semantic memory.. Journal of Verbal Learning and Verbal Behavior.

[9] Steyvers M, Tenenbaum JB (2005). The large-scale structure of semantic networks: statistical analyses and a model of semantic growth.. Cognitive Science.

[10] Newman MEJ (2001). Clustering and preferential attachment in growing networks.. Physical Review E.

[11] Hills TT, Maouene M, Maouene J, Sheya A, Smith LB (2009). Longitudinal analysis of early semantic networks: preferential attachment or preferential acquisition?. Psychological Science.

[12] Hills TT, Maouene J, Riordan B, Smith LB (2010). The Associative Structure of Language: Contextual Diversity in Early Word Learning.. Journal of memory and language.

[13] Watts DJ, Strogatz SH (1998). Collective dynamics of “small-world” networks.. Nature.

[14] Hills TT, Maouene M, Maouene J, Sheya A, Smith LB (2009). Categorical structure among shared features in networks of early-learned nouns.. Cognition.

[15] Banavar JR, Maritan A, Rinaldo A (1999). Size and form in efficient transportation networks.. Nature.

[16] Bates E, Marchman V, Thal DJ, Fenson L, Dale PS (1994). Developmental and stylistic variation in the composition of early vocabulary.. Journal of child language.

[17] Dupuy H (1974). The rationale, development and standardization of a basic word vocabulary test..

[18] Fenson L, Dale PS, Reznick JS, Thal DJ, Bates E (2002). MacArthur Communicative Development Inventories: User's guide and technical manual..

[19] Thal DJ, Bates E, Goodman J, Jahn-Samilo J (1997). Continuity of language abilities: An exploratory study of late-and early-talking toddlers.. Developmental neuropsychology,.

[20] Anderson RC, Freebody P, Guthrie J (1985). Vocabulary knowledge.. Theoretical models and processes of reading.

[21] Moyle MJ, Ellis Weismer S, Evans JL, Lindstrom MJ (2007). Longitudinal relationships between lexical and grammatical development in typical and late-talking children..

[22] Rescorla L (2002). Language and reading outcomes to age 9 in late-talking toddlers.. Journal of Speech, Language, and Hearing Research.

[23] Weismer SE (2006). Typical Talkers, Late Talkers, and Children With Specific Language Impairment: A Language Endowment Spectrum? Language disorders from a developmental perspective: Essays in honor of Robin S.. Chapman: 83.

[24] Zubrick SR, Taylor CL, Rice ML, Slegers DW (2007). Late language emergence at 24 months: An epidemiological study of prevalence, predictors, and covariates.. Journal of Speech, Language, and Hearing Research.

[25] Dale PS, Bates E, Reznick JS, Morisset C (1989). The validity of a parent report instrument of child language at twenty months.. Journal of child language,.

[26] Dale PS, Fenson L (1996). Lexical development norms for young children.. Behavior Research Methods Instruments and Computers.

[27] MacWhinney B (1995). The CHILDES project: Tools for analyzing talk..

[28] Hofmann T (1999). Probabilistic latent semantic indexing..

[29] Li P, Farkas I, MacWhinney B (2004). Early lexical development in a self-organizing neural network.. Neural Networks.

[30] Boccaletti S, Latora V, Moreno M, Chavez M, Hwang D (2006). Complex networks: structure and dynamics.. Physics Report.

[31] Erdös P, Rényi A (1960). On the evolution of random graphs.. Publications of the Mathematical Institute of the Hungarian Academy of Sciences.

[32] Barabási AL, Albert R (1999). Emergence of scaling in random networks.. Science.

[33] Vigil DC, Hodges J, Klee T (2005). Quantity and quality of parental language input to late-talking toddlers during play.. Child Language Teaching and Therapy.

